# Prefrontal network engagement by deep brain stimulation in limbic hubs

**DOI:** 10.3389/fnhum.2023.1291315

**Published:** 2024-01-12

**Authors:** Anusha Allawala, Kelly R. Bijanki, Denise Oswalt, Raissa K. Mathura, Joshua Adkinson, Victoria Pirtle, Ben Shofty, Meghan Robinson, Matthew T. Harrison, Sanjay J. Mathew, Wayne K. Goodman, Nader Pouratian, Sameer A. Sheth, David A. Borton

**Affiliations:** ^1^School of Engineering, Brown University, Providence, RI, United States; ^2^Department of Neurological Surgery, University of California, San Francisco, San Francisco, CA, United States; ^3^Department of Neurosurgery, Baylor College of Medicine, Houston, TX, United States; ^4^Department of Neurosurgery, University of Pennsylvania, Philadelphia, PA, United States; ^5^Department of Neurosurgery, University of Utah, Salt Lake City, UT, United States; ^6^Division of Applied Mathematics, Brown University, Providence, RI, United States; ^7^Menninger Department of Psychiatry and Behavioral Sciences, Baylor College of Medicine, Houston, TX, United States; ^8^Department of Neurological Surgery, UT Southwestern Medical Center, Dallas, TX, United States; ^9^Department of Veterans Affairs, Center for Neurorestoration and Neurotechnology, Providence, RI, United States

**Keywords:** deep brain stimulation (DBS), major depressive disorder (MDD), ventral capsule/ventral striatum, subcallosal cingulate, gamma oscillations, prefrontal networks, stereo-EEG/intracranial recordings

## Abstract

Prefrontal circuits in the human brain play an important role in cognitive and affective processing. Neuromodulation therapies delivered to certain key hubs within these circuits are being used with increasing frequency to treat a host of neuropsychiatric disorders. However, the detailed neurophysiological effects of stimulation to these hubs are largely unknown. Here, we performed intracranial recordings across prefrontal networks while delivering electrical stimulation to two well-established white matter hubs involved in cognitive regulation and depression: the subcallosal cingulate (SCC) and ventral capsule/ventral striatum (VC/VS). We demonstrate a shared frontotemporal circuit consisting of the ventromedial prefrontal cortex, amygdala, and lateral orbitofrontal cortex where gamma oscillations are differentially modulated by stimulation target. Additionally, we found participant-specific responses to stimulation in the dorsal anterior cingulate cortex and demonstrate the capacity for further tuning of neural activity using current-steered stimulation. Our findings indicate a potential neurophysiological mechanism for the dissociable therapeutic effects seen across the SCC and VC/VS targets for psychiatric neuromodulation and our results lay the groundwork for personalized, network-guided neurostimulation therapy.

## 1 Introduction

The ability to regulate complex emotions and make controlled decisions are central to the human experience and critical for successful navigation through challenging life circumstances. Neuroimaging and electrodiagnostic studies have implicated prefrontal networks encompassing the dorsal anterior cingulate cortex (dACC), orbitofrontal cortex (OFC), ventromedial prefrontal cortex (vmPFC) and amygdala in affective and emotional regulation (Delgado et al., 2008; Etkin et al., 2011, 2015; Groenewold et al., 2013; Hiser and Koenigs, 2018), decision making and impulsivity (Elliott et al., 2000; Shenhav et al., 2013; Hiser and Koenigs, 2018), reward evaluation (Bush et al., 2002; Lipsman et al., 2014; Saez et al., 2018; Knudsen and Wallis, 2020), and emotional processing (Pessoa and Adolphs, 2010; Geissberger et al., 2020). Within electrophysiology studies spanning across species, both low and high frequency oscillations across prefrontal, limbic and cingulate structures have emerged as key signals involved in distinct aspects of cognitive, affective and reward processing. Examples of such signals include theta band (4–8 Hz) and gamma band activity (60–140) in the dACC (Rothé et al., 2011; Heilbronner and Hayden, 2016; Widge et al., 2019) for cognitive control processing and adaptation, respectively, alongside gamma activity for conflict processing in the OFC (Tang et al., 2016). Similarly, theta, beta (13–35 Hz) and gamma activity have emerged as critical neural features representing reward valuation, expectation, modulation and processing the OFC (van Wingerden et al., 2010; Sacré et al., 2016; Saez et al., 2018; Knudsen and Wallis, 2020; Amarante and Laubach, 2021). These spectral features are also modulated in the vmPFC and dACC during affective processing (Lipsman et al., 2014; Bijanzadeh et al., 2022). Of note, depending on anatomical structure and their associated role in executive, affective or reward function, some distinct spectral features have shown to be replicable across species and some studies (e.g., midfrontal and cingulate theta oscillations in cognitive control function). Disruption of neural activity in these implicated circuits is thought to lead to psychiatric disorders of mood, anxiety, and impulsivity, among other behavioral manifestations (Price and Drevets, 2010; Groenewold et al., 2013; Cheng et al., 2016; Williams, 2016; Ferri et al., 2017; Damborská et al., 2020; Rolls et al., 2020). Further, causal manipulations of networks underlying regulation of emotional and cognitive processing using electrical stimulation and lesioning have provided further evidence of the close relationship between disrupted neural circuits and behavioral symptoms in psychiatric disorders (Drevets, 2007; Wilson et al., 2014; Heilbronner et al., 2016; Schneider and Koenigs, 2017; Basu et al., 2019; Sawada et al., 2022). Neuromodulatory interventions (Mayberg et al., 2005; Scangos et al., 2021a) are often used to treat such disorders, but little is known about the human *electrophysiology* of these prefrontal regions in psychiatric disorders and how chronic neurostimulation therapies *modify* circuit dynamics underlying psychiatric symptoms. Characterizing the specific spatiotemporal prefrontal network activity implicated in affective and cognitive processing in response to therapeutic stimulation can inform stimulation paradigms on a chronic or adaptive basis and aid the prediction of an individual’s response to stimulation.

Two well-characterized affective hubs previously demonstrated to be gateways to parsimoniously engage prefrontal and corticolimbic networks through invasive means (Mayberg et al., 2005; Quraan et al., 2014; Widge et al., 2019; Elias et al., 2022) are the ventral capsule/ventral striatum (VC/VS) and subcallosal cingulate (SCC). The VC/VS and SCC are thought to be hubs (Crowell et al., 2014) at the crossroads of white matter pathways hypothesized to influence executive function (Widge et al., 2019), reward processing (Rogers et al., 2004; Heldmann et al., 2012) and mood processing (Mayberg et al., 2005; Malone et al., 2009) through their connections of varying degrees to prefrontal and limbic structures (spanning the amygdala, PFC and ACC) (Haber, 2012; Heilbronner and Haber, 2014) with partial overlap (Gutman et al., 2009; Riva-Posse et al., 2014; Zhu et al., 2021). Modulation of the two targets have shown promising results in DBS studies showing improvement in symptoms of anxiety (Lipsman et al., 2013), depression (Mayberg et al., 2005; Holtzheimer et al., 2012; Ramasubbu et al., 2013), treatment-refractory anorexia nervosa (Lipsman et al., 2017), addiction (Mantione et al., 2010; Voges et al., 2013; Kuhn et al., 2014) and obsessive compulsive disorder (Smith et al., 2020; van der Vlis et al., 2021). In the clinical treatment of treatment-resistant depression (TRD) with deep brain stimulation (DBS), the VC/VS and SCC targets both showed initially promising open-label studies (Mayberg et al., 2005; Malone et al., 2009). However, these studies were followed up by controlled trials that failed to meet sufficient outcomes measures ultimately needed for regulatory use of DBS for treatment refractory depression (Dougherty et al., 2015; Holtzheimer et al., 2017). Of interest, while both targets can have an antidepressant effect, responses to stimulation across the two targets are phenotypically different, and notable qualitative differences in behavioral responses (“activating” vs. “calming”) (Mayberg et al., 2005; Malone et al., 2009; Choi et al., 2015; Scangos et al., 2021b; Sheth et al., 2021) have been observed. Prefrontal targets appear to be key in driving a response from both DBS targets (Brown et al., 2020; Clark et al., 2020; Liebrand et al., 2020).

The aim of our study was to evaluate the network-level effects of acute stimulation *between* the SCC and VC/VS, two well-established targets used for psychiatric DBS therapy. We took advantage of a unique opportunity afforded through an ongoing clinical trial of DBS for TRD (NCT03437928) where we performed acute stimulation experiments using segmented DBS leads in the SCC and VC/VS with concurrent high-density intracranial recordings providing high spatiotemporal resolution of neural activity in two participants with TRD. We aimed to characterize the *differences* in neural response across prefrontal networks between the two DBS targets within and across patients with TRD. Given the phenotypic differences that are observable following stimulation of the VC/VS and SCC, and established role of low and high oscillations in the vmPFC, OFC, dACC and amygdala in neuropsychiatric disorders (Drevets, 2007; Myers-Schulz and Koenigs, 2012; Ferri et al., 2017; Rao et al., 2018; McTeague et al., 2020), we hypothesized that we would find differentiable neurophysiological responses to acute stimulation between the DBS targets in our aforementioned regions of interest (OFC, vmPFC, dACC, amygdala) that this would be unique to anatomical regions in high frequency activity (defined as 13–100 Hz for this study) or low frequency activity (defined as 1–13 Hz for our study) building on recent work implicating frequency-specific neural oscillations in mood (Lipsman et al., 2014; Rao et al., 2018; Scangos et al., 2021a; Bijanzadeh et al., 2022). Our results lay the groundwork for a more mechanistic understanding of the effects of DBS across prefrontal circuits in psychiatric disease, and better equip us to implement optimized, network-guided neuromodulation in the future.

## 2 Materials and methods

### 2.1 Participant and study overview

Data for this study was collected from two participants (37 year old Latino male and a 57 year old Caucasian female) diagnosed with TRD. The participants were enrolled in an ongoing clinical trial (NCT 03437928) for DBS for TRD. Each participant gave fully informed consent according to study sponsor guidelines, and all procedures were approved by the local institutional review board at Baylor College of Medicine IRB (H-43036) prior to participation. The trial has enrolled more than two participants, but due to the changing nature of the goals of the study, certain aspects of the stimulation experiments have changed across participants. In particular, the stimulation paradigm (described below in section “2.3 Electrode stimulation and recording”) for the first two participants changed such that the analyses described here were not possible in subsequent participants. Rather than combining heterogeneous analyses, we focused on the data from these first two participants with consistent acquired data.

Participants underwent stereotactic implantation of four DBS leads (Boston Scientific Cartesia, Marlborough, MA, USA) and 10 temporary sEEG electrodes (PMT, Chanhassen, MN, USA) based on pre-operative scans including patient-specific tractography. Post-implantation, patients underwent a 10-day intracranial monitoring period for evaluation of brain networks involved in depression. Following the intracranial monitoring period, sEEG electrodes were removed and the four DBS leads were internalized and connected to two implanted pulse generators (IPG) (Boston Scientific Gevia, Marlborough, MA, USA). Additional surgical details have been described previously (Sheth et al., 2021).

### 2.2 Electrode implantation

**FIGURE 1:**
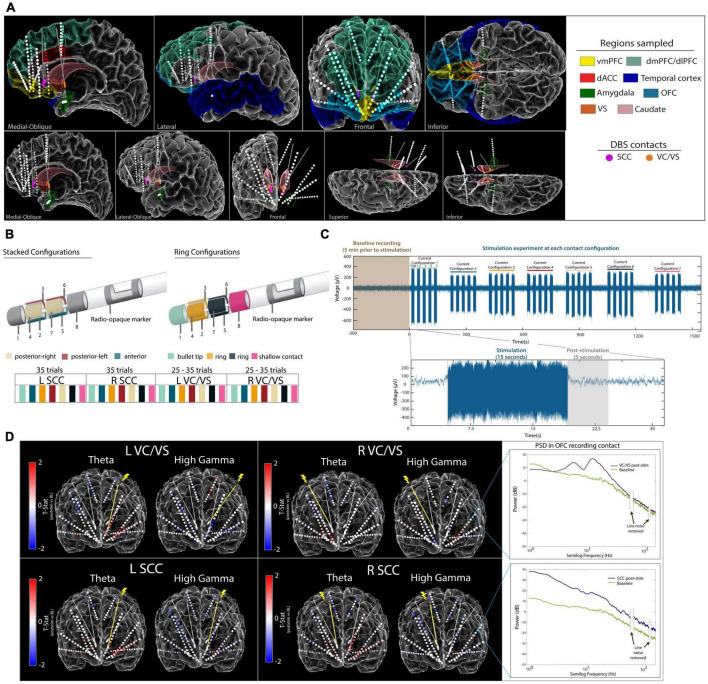
Experimental Approach. **(A)** Anatomical reconstruction showing placement of sEEG electrodes (top panel) and DBS leads (bottom panel). Colors in the legend (right) correspond to the region where electrodes were implanted. The vmPFC, OFC, dACC, and Amygdala were regions of interest for this study. **(B)** Steerable DBS leads were used to deliver unilateral stimulation in the VC/VS and SCC, respectively. Seven current configurations of interest were identified and tested across participants. **(C)** Raw voltage signal recorded on an example sEEG contact during stimulation in the left SCC DBS lead. Participants were systematically tested at each current configuration for 15 s, with five trials per current configuration at each respective DBS lead. A 5-s window following stimulation was used for subsequent analyses.**(D)** Electrode diagram showing the mean power change in theta band and high gamma band following stimulation for participant A. Each contact is colored based on the t-statistic value computed between baseline and post-stimulation for each DBS lead (red indicates increase in power following stimulation and blue indicates a decrease in power following stimulation). Inset (right) shows log-transformed power spectra from a recording electrode in the OFC during baseline and post-stimulation. vmPFC, ventromedial prefrontal cortex; lOFC, lateral orbitofrontal cortex; mOFC, medial orbitofrontal cortex; dACC, dorsal anterior cingulate cortex; VC/VS, ventral capsule/ventral striatum; SCC, subcallosal cingulate.

Intracranial sEEG electrodes for local field potential (LFP) recordings were implanted bilaterally across several cortical and subcortical targets based on previous work implicating their roles in mood, reward, as well as cognitive and affective processing (Miller, 2000; Etkin et al., 2015; Knudsen and Wallis, 2020; Friedman and Robbins, 2021). Regions sampled included the dorsolateral prefrontal cortex (dlPFC), ventromedial prefrontal cortex (vmPFC), dorsal anterior cingulate cortex (dACC), lateral and medial orbitofrontal cortex (lOFC, mOFC), superior frontal gyrus (SFG), superior and medial temporal gyri (STG, MTG) and the amygdala (Figure 1A). Post-operative CT scans and pre-operative MRI scans were aligned using the Functional Magnetic Resonance Imaging for the Brain Software Library’s (FMRIB’s) Linear Image Registration Tool (FLIRT). Electrode coordinates were manually determined from the co-registered CT in BioImage Suite and placed into native MRI space. The reconstructed cortical surface, segmented cortical and subcortical structures and electrode coordinates were visualized using the Multi-Modal Visualization Tool (Felsenstein et al., 2019).

Both participants were implanted bilaterally with segmented DBS leads in the VC/VS and the SCC, capable of current steering. The DBS leads used in our study consist of eight stimulation contacts: solid ring contacts at the deepest and shallow positions, as well as three-way segmented contacts located between the ring contacts. Seven total contact configurations of interest were identified per lead, including three stacked configurations listed as follows: (1) anterior-facing contacts 2 and 5, (2) posterior-left facing contacts 4 and 7 and (3) posterior-right facing contacts 3 and 6. The remaining four configurations tested were ring configurations listed as follows: (1) solid ring contact 1 (2) solid ring contact 8 (3) combination of segmented ring contacts 2, 3, and 4, and (4) combination of segmented ring contacts 5, 6, and 7 (Figure 1B).

### 2.3 Electrode stimulation and recording

Monopolar cathodic stimulation was delivered through each DBS lead via a Blackrock CereStim R96 (Blackrock Microsystems, Salt Lake City, UT). A stimulation amplitude of 4.8–5 mA was delivered at the solid ring contacts, whereas for stacked or ring configurations this amplitude was split evenly among contacts to enable current steering, never exceeding 5 mA in total or at any time a charge density of 30 μC/cm^2^. Stimulation was applied at 130 Hz, with a pulse width of 180 μS and interphase gap of 100 μS. In participant A, we tested the seven identified stimulation combinations (3 stacked configurations, 4 ring configurations) for each DBS lead in the SCC, and five combinations (3 stacked configurations, 2 ring configurations) in each DBS lead in the VC/VS. In participant B, we tested all seven combinations across each of the four DBS leads. Each trial of stimulation consisted of 15 s of stimulation on followed by 10 s without stimulation (Figure 1C). Trials were repeated 5 times per contact configuration per DBS lead seriatim, resulting in 25–35 trials per DBS lead for participant A and 35 trials per DBS lead for participant B.

### 2.4 Data acquisition and signal processing

Electrophysiological signals from implanted sEEG electrode contacts were recorded using a 256-channel NeuroPort Acquisition System (Blackrock Microsystems, UT, USA) at a sampling rate of 2 kHz, with a hardware high pass filter applied at 0.3 Hz. Recordings from sEEG contacts were analyzed offline using custom scripts written in MATLAB (Mathworks Inc. Natick, MA, USA) and Python. LFP signals were demeaned, decimated to 1 kHz and bandpass filtered between 1 and 250 Hz. A butterworth notch filter was applied to remove line noise at 60, 120, and 180 Hz, respectively. Recordings were bipolar re-referenced by subtracting the activity of adjacent electrode contact pairs. Any channels with excessive noise or without a clear neural signal were removed from the analysis. To evaluate the response of the sampled networks before and after stimulation, we analyzed the spectral power in the 5 s following stimulation to avoid artifact contamination. We identified a window of 600 ms post-stimulation that was additionally excluded from analysis to avoid residual post-stimulation artifacts in the signal. The multitaper spectral estimation method was used to extract power spectral density (PSD) from the sEEG recording using the *mspectrumc.m* function from the Chronux toolbox (Bokil et al., 2010). Spectral power was then averaged within standard frequency bands (delta = 1–4 Hz, theta = 4–8 Hz, alpha = 8–12 Hz, beta = 12–30 Hz, low-gamma = 30–55 Hz, high-gamma = 65–100 Hz). The spectral power across the baseline windows and post-stimulation windows across all stimulation experiments were z-scored within participants. Electrode contacts in gray matter located in the regions of interest were identified and pre-processed signals were then grouped (averaged) per region of interest. The location of electrode contacts in gray matter vs. white matter and the labels for anatomical region of interest was verified by visual review of the MRI and CT by an expert rater (BS).

Prior to starting the stimulation experiments, 5 min of baseline recording was collected for each participant (Figure 1C). To avoid temporal autocorrelation, the autocorrelation was computed for spectral power within each frequency band of interest and region of interest, across time (Supplementary Figure 2). The number of lags *t* where the autocorrelation was at or below 0.1 and all subsequent autocorrelation values were between 0.1 and −0.1 was identified. Lag *t* was then used to generate surrogate “trials” from the baseline recording, where *t* seconds was skipped every 5 s. The resulting 5-s trials were used for analysis to compare against the post-stimulation windows during the stimulation experiments (additional details in Supplementary Figure 2).

### 2.5 Statistical analysis

As this study includes two participants, no conclusions about a clinical population with refractory depression can be drawn. The goal with the described analyses is to identify robust, statistically reliable patterns of stimulation-induced network response observed *within* a given participant that cannot be explained by random variation or chance. In order to carefully control for multiple comparisons as our analyses is performed to assess differences across conditions (pre- and post- stimulation, VC/VS vs. SCC stimulation) across ROIs and neural features, we performed the statistical testing procedures described below.

To test the difference between pre- (baseline) and post-stimulation, non-parametric permutation testing was performed on the z-scored data using custom scripts written in MATLAB. Data labels from post-stimulation and baseline windows were randomly shuffled, and then the absolute value of the t-statistic for a two-sample, pooled variance, *t*-test was computed for each pair of shuffled data. This procedure was repeated 1000 times. We used a single-step maxT procedure to correct for multiple tests (Westfall and Stanley Young, 1993; Nichols and Holmes, 2002), namely, each absolute t-statistic was compared to the distribution (over permutations) of the maximal absolute t-statistic across all regions of interest and frequency bands of interest in order to obtain corrected *p*-values that control the familywise error rate (corrected *p*-values reported in Supplementary Tables 1–4 and uncorrected *p*-values are reported in Supplementary Tables 7–10).

To test the difference between PSD changes across the network following unilateral VC/VS stimulation vs. SCC stimulation, non-parametric permutation testing was performed on the z-scored data using custom scripts written in MATLAB. We compared the effect of unilateral VC/VS to unilateral SCC stimulation (i.e., left VC/VS was tested against left SCC stim and right VC/VS stim was tested against right SCC stim). Data labels for unilateral SCC stimulation and unilateral VC/VS stimulation were randomly shuffled, and then the absolute value of the t-statistic for a two-sample, pooled variance, *t*-test was computed for each pair of shuffled data. This procedure was repeated 1000 times. We used a single-step maxT procedure to correct for multiple tests (Westfall and Stanley Young, 1993; Nichols and Holmes, 2002), namely, each absolute t-statistic was compared to the distribution (over permutations) of the maximal absolute t-statistic across all regions of interest and frequency bands of interest in order to obtain corrected *p*-values that control the familywise error rate (corrected *p*-values reported in Supplementary Tables 5, 6 and uncorrected *p*-values are reported in Supplementary Tables 11, 12). Additional details on statistical testing are described in the Supplementary material.

## 3 Results

The goal of our study was to quantify prefrontal network responses to intracranial stimulation between two DBS targets: the SCC and VC/VS. We first evaluated the effects of stimulation for each DBS target (pre- vs. post-stim, *p*-values adjusted to compensate for multiple comparisons reported in Supplementary Methods and Supplementary Tables 1–4; Figure 1C) on high-density stereo-EEG (sEEG recordings) in two participants with TRD (Figures 1A, B and Supplementary Methods). We then compared neural responses (see Supplementary Methods) following stimulation between the two DBS targets (SCC post-stim vs. VC/VS post-stim, adjusted *p*-values reported in Supplementary Tables 5, 6) on high frequency neural activity (beta, low gamma and high gamma band power) and low frequency activity (delta, theta and alpha band power). A representative example of the electrode coverage is shown in Figure 1D, illustrating bilateral modulation of low frequency power (e.g., theta) and high frequency power (e.g., high gamma) across recording contacts following unilateral stimulation in participant A. Statistical limitations in our study with *N* = 2 participants preclude any conclusions about a broader clinical population. However, identifying differences across conditions *within* a participant that cannot be estimated by chance entails carefully accounting for the multiple testing problem as we have done in our statistical analyses described in the section “2 Materials and methods.”

Given the previously established roles of the dACC, amygdala, OFC and vmPFC in affective and cognitive regulation in psychiatric disorders, we focused our analyses across these key four anatomical regions within each subject and describe our findings for each key region in detail below.

### 3.1 vmPFC

**FIGURE 2:**
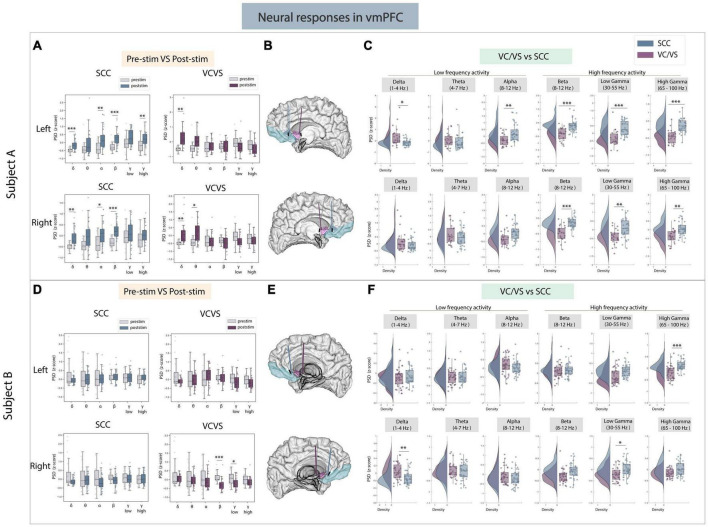
Neural responses in the vmPFC following SCC stimulation vs. VC/VS stimulation. **(A)** Distribution of spectral power across all post-stimulation trials vs. pre-stimulation (baseline) in the vmPFC after z-scoring in six pre-defined frequency bands (delta, theta, alpha, beta, low gamma, and high gamma) following SCC stimulation (left) and VC/VS stimulation (right) in participant A **(B)** Corresponding anatomical location of the vmPFC highlighted in light blue and corresponding VC/VS and SCC DBS leads highlighted depending on hemisphere of stimulation. Stimulation in left hemisphere is on top, while stimulation in right hemisphere is shown on the bottom. **(C)** Distribution of spectral power across six pre-defined frequency bands contrasting neural responses following SCC stimulation and VC/VS stimulation. **(D–F)** Replicate of figures in panels **(A–C)** for participant B. *Indicates significance where adj.*p*-value < 0.05, corrected; **Indicates significance where adj.*p*-value ≤ 0.01, corrected; ***Indicates significance, where adj.*p*-value ≤ 0.001, corrected.

We first sought to understand the effect of stimulation on high frequency activity in the vmPFC given its broad involvement in cognitive, affective and emotional processing (Hiser and Koenigs, 2018), shown in Figures 2A–F. Here, we found consistent differences when evaluating neural responses in high frequency bands between SCC and VC/VS stimulation (Figures 2C, F) in both participants. **Specifically, we found that SCC consistently increased gamma power in both participants while VC/VS decreased gamma power.** Within Participant A, left SCC stimulation elicited a significant increase in spectral power in high gamma (pre- vs. post-stim, adj.*p* < 0.01). The response to stimulation was significantly different between both DBS targets in high gamma band (adj.*p* < 0.01) and low gamma band (adj.*p* < 0.01) irrespective of the hemisphere of stimulation. The inverse relationship in which SCC increased high-frequency activity and VC/VS decreased high-frequency activity was also observed in beta band (adj.*p* < 0.001) in participant A. In Participant B (Figure 2D), we observed right VC/VS stimulation significantly decreased low gamma power (adj.*p* < 0.05) and beta power (adj.*p* < 0.001) from baseline. In the same participant, neural responses were significantly different between the two DBS targets as observed in low gamma (adj.*p* < 0.05) and high gamma (adj.*p* < 0.01; Figure 2F).

We next explored if the same opposing response between SCC and VC/VS was observed in low-frequency activity. We did observe a significant difference in delta power between SCC and VC/VS stimulation in both participants (Figures 2C, F). While both SCC and VC/VS stimulation significantly increased delta power from baseline (adj.*p* < 0.01), respectively, we found that VC/VS stimulation drove a larger increase in delta power than SCC stimulation and the responses between the two DBS targets were significantly different (adj.*p* < 0.05) in participant A. In participant B, it appears that while both SCC and VC/VS stimulation drive a decrease in delta power from baseline (Figure 2D), the response between the two targets is still significantly different, where VC/VS drives a smaller decrease than SCC stimulation (adj.*p* < 0.01; Figure 2F).

### 3.2 Amygdala

**FIGURE 3:**
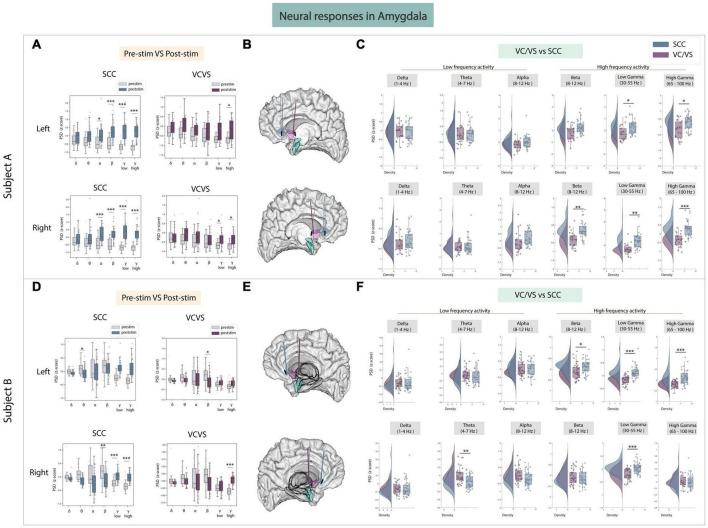
Neural responses in the amygdala following SCC stimulation vs. VC/VS stimulation. **(A)** Distribution of spectral power across all post-stimulation trials vs. pre-stimulation (baseline) in the amygdala after z-scoring in six pre-defined frequency bands (delta, theta, alpha, beta, low gamma, and high gamma) following SCC stimulation (left) and VC/VS stimulation (right) in participant A **(B)** Corresponding anatomical location of the amygdala highlighted in green and corresponding VC/VS and SCC DBS leads highlighted depending on hemisphere of stimulation. Stimulation in left hemisphere is on top, while stimulation in right hemisphere is shown on the bottom. **(C)** Distribution of spectral power across six pre-defined frequency bands contrasting neural responses following SCC stimulation and VC/VS stimulation. **(D–F)** Replicate of figures in panels **(A–C)** for participant B. *Indicates significance where adj.*p*-value < 0.05, corrected; **Indicates significance where adj.*p*-value ≤ 0.01, corrected; ***Indicates significance, where adj.*p*-value ≤ 0.001, corrected.

The next area of interest for this study was the amygdala (Figures 3A–F), given its role in emotional regulation (Pessoa and Adolphs, 2010). In the amygdala, **while both VC/VS and SCC stimulation elicited increases in low and high gamma power, responses in both low and high gamma were still significantly different between the DBS targets in both participants** (Figures 3C, F). SCC stimulation significantly increased high frequency activity from baseline in both participants (Figures 3A, D). In participant A, SCC stimulation drove a significant increase in beta (adj.*p* < 0.001), low gamma (adj.*p* < 0.001) and high gamma power (adj.*p* < 0.001). In participant B, right SCC stimulation significantly increased low gamma (adj.*p* < 0.001) and high gamma power (adj.*p* < 0.001) while significantly decreasing beta power (adj.*p* < 0.01). VC/VS stimulation also significantly increased high gamma power in both participants (adj.*p* < 0.05; Figures 3A, D). In participant A, right VC/VS stimulation also significantly increased low gamma power (adj.*p* < 0.05) and in participant B, left VC/VS stimulation significantly decreased beta power (adj.*p* < 0.05). When contrasting neural responses between the two DBS targets (Figures 3C, F) we observed that SCC increased low gamma (adj.*p* < 0.05) and high gamma power (adj.*p* < 0.05) significantly higher than VC/VS stimulation, and right SCC increased beta power significantly higher than VC/VS stimulation (adj.*p* < 0.01) in participant A. In participant B, beta power and high gamma power were again significantly higher (adj.*p* < 0.05) following right SCC stimulation compared to right VC/VS stimulation, and low gamma power was significantly higher (adj.*p* < 0.001) following SCC stimulation compared to VC/VS stimulation irrespective of the hemisphere of stimulation.

Across low frequency bands, we found responses to SCC and VC/VS stimulation were significantly different in theta band in participant B (adj.*p* < 0.01; Figures 3C, F). Here, SCC drove a decrease in power relative to the VC/VS. However, no consistent modulation of low frequency activity across the two participants was otherwise observed.

### 3.3 Lateral and medial OFC

**FIGURE 4:**
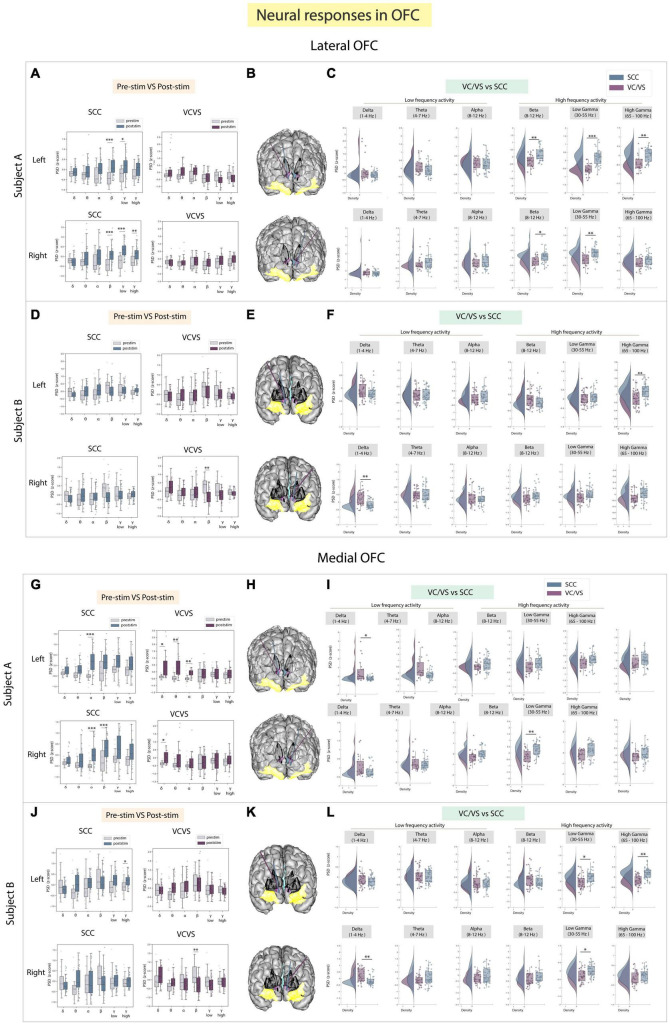
Neural responses in the OFC following SCC stimulation vs. VC/VS stimulation. **(A)** Distribution of spectral power across all post-stimulation trials vs. pre-stimulation (baseline) in the lOFC after z-scoring in six pre-defined frequency bands (delta, theta, alpha, beta, low gamma, and high gamma) following SCC stimulation (left) and VC/VS stimulation (right) in participant A **(B)** Corresponding anatomical location of OFC highlighted in yellow and corresponding VC/VS and SCC DBS leads highlighted depending on hemisphere of stimulation. Stimulation in left hemisphere is on top, while stimulation in right hemisphere is shown on the bottom. **(C)** Distribution of spectral power across six pre-defined frequency bands contrasting neural responses following SCC stimulation and VC/VS stimulation. **(D–F)** Replicate of figures in panels **(A–C)** for participant B. **(G)** Distribution of spectral power across all post-stimulation trials vs. pre-stimulation (baseline) in the mOFC after z-scoring in six pre-defined frequency bands following SCC stimulation (left) and VC/VS stimulation (right) in participant A. **(H)** Corresponding anatomical location of OFC highlighted in yellow and corresponding VC/VS and SCC DBS leads highlighted depending on hemisphere of stimulation. Stimulation in left hemisphere is on top, while stimulation in right hemisphere is shown on the bottom. **(I)** Distribution of spectral power across six pre-defined frequency bands contrasting neural responses following SCC stimulation and VC/VS stimulation. **(J–L)** Replicate of figures in panels **(G–I)** for participant B. *Indicates significance where adj.*p*-value < 0.05, corrected; **Indicates significance where adj.*p*-value ≤ 0.01, corrected; ***Indicates significance, where adj.*p*-value ≤ 0.001, corrected.

The lOFC was a third target of interest because it has been implicated in cognitive and reward processing and recently employed as a target for neuromodulation to improve mood (Rao et al., 2018; Scangos et al., 2021b). Results to stimulation between DBS targets are shown in Figures 4A–F. We found neural responses to SCC stimulation were significantly different from VC/VS stimulation in participant A (adj.*p* < 0.05; Figure 4C) and once again followed the same inverse relationship seen in the amygdala, **where SCC drove an increase in power in beta, low gamma, and high gamma bands relative to the VC/VS.** In the same participant, SCC stimulation also significantly increased beta, low gamma and high gamma power from baseline (adj.*p* < 0.01). In participant B, the significant difference in response between SCC and VC/VS stimulation was observed in high gamma power (adj.*p* < 0.01; Figure 4F) following stimulation in the left hemisphere. Surprisingly, we did not find many differences in neural responses to SCC and VC/VS stimulation across lower frequency bands. Delta band power was significantly modulated, (adj.*p* < 0.01): right VC/VS stimulation increased delta power and right SCC stimulation decreased delta power. The differential delta band power change was, however, participant specific.

We explored stimulation response in the mOFC separately as the lateral and medial orbitofrontal structures have shown to have distinct roles in cognitive and reward processing (Elliott et al., 2000). In the mOFC (Figures 4G–L), we found that differences between responses in high frequency activity following SCC vs. VC/VS stimulation were participant specific in the mOFC (Figures 4I, L). For example, the inverse relationship where SCC increases high frequency activity and VC/VS decreases high frequency activity was observed in participant A. Here, right SCC stimulation significantly increased beta power from baseline (adj.*p* < 0.001; Figure 4G), and we observed a significant difference in response to stimulation in beta power between the SCC and the VC/VS (adj.*p* < 0.01, Figure 4I). In participant B, SCC significantly increased high gamma power from baseline (adj.*p* < 0.05; Figure 4J). The inverse relationship between SCC and VC/VS stimulation-induced responses of high frequency activity was observed in low gamma (adj.*p* < 0.05) and high gamma (adj.*p* < 0.01), where SCC stimulation increased activity relative to VC/VS stimulation (Figure 4L).

When assessing stimulation response in low frequency activity in the mOFC, we found significant differences between responses to VC/VS vs. SCC stimulation seen across both participants in delta band (Figures 4I, L). In participant A, left VC/VS stimulation significantly increased power in delta band, and this response was significantly higher than the neural response following SCC stimulation (adj.*p* < 0.05). In participant B, right VC/VS stimulation significantly increased delta power relative to SCC stimulation (adj.*p* < 0.01).

### 3.4 dACC

**FIGURE 5:**
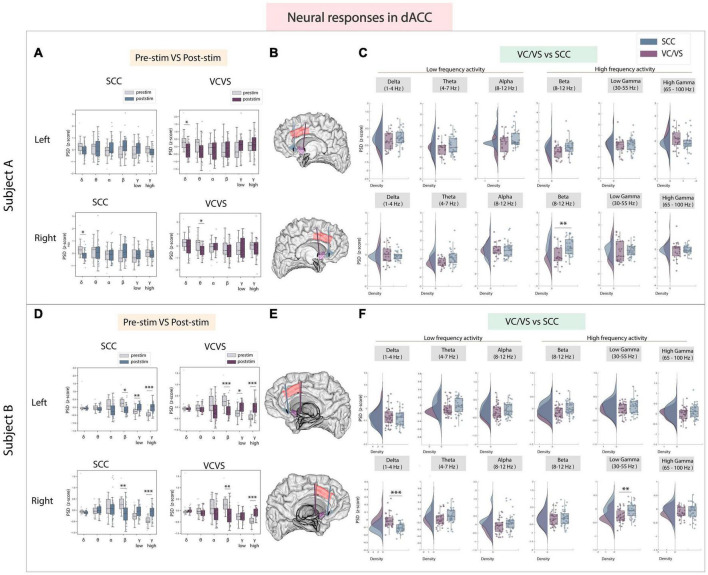
Neural responses in the dACC following SCC stimulation vs. VC/VS stimulation. **(A)** Distribution of spectral power across all post-stimulation trials vs. pre-stimulation (baseline) in the dACC after z-scoring in six pre-defined frequency bands (delta, theta, alpha, beta, low gamma, and high gamma) following SCC stimulation (left) and VC/VS stimulation (right) in participant A **(B)** Corresponding anatomical location of dACC highlighted in red and corresponding VC/VS and SCC DBS leads highlighted depending on hemisphere of stimulation. Stimulation in left hemisphere is on top, while stimulation in right hemisphere is shown on the bottom. **(C)** Distribution of spectral power across six pre-defined frequency bands comparing neural responses between SCC stimulation and VC/VS stimulation. **(D–F)** Replicate of figures in panels **(A–C)** for participant B. *Indicates significance where adj.*p*-value < 0.05, corrected; **Indicates significance where adj.*p*-value ≤ 0.01, corrected; ***Indicates significance, where adj.*p*-value ≤ 0.001, corrected.

A key region known to play an important role in cognitive control and emotional processing is the dACC (Shenhav et al., 2013; Etkin et al., 2015). In the dACC (Figures 5A–F), when examining high frequency activity, we found that significant differences following stimulation between the SCC and VC/VS were also individual-specific in the dACC but still followed the inverse relationship between the two DBS targets observed in high frequency activity in other ROIs (Figures 5C, F). Right SCC stimulation significantly increased beta power (adj.*p* < 0.01) compared to VC/VS stimulation in participant A. In participant B, left SCC stimulation significantly increased low gamma power from baseline (adj.*p* < 0.01) while stimulation of either hemisphere in the SCC increased high gamma power (adj.*p* < 0.001; Figure 5D). Left VC/VS stimulation similarly significantly increased low gamma power (adj.*p* < 0.05) while stimulation of either hemisphere significantly increased high gamma power from baseline (adj.*p* < 0.01; Figure 5D). However, the responses between left SCC and left VC/VS stimulation were still significantly different in low gamma band (adj.*p* < 0.01; Figure 5F) and SCC stimulation drove a larger increase in low gamma power relative to the VC/VS.

When examining low frequency activity in response to stimulation in the dACC, we also observed a significant difference between VC/VS and SCC stimulation in delta power (adj.*p* < 0.001) in participant B–similar to the pattern observed in other ROIs–where SCC decreased power in a low frequency band (delta) and VC/VS stimulation increased power. In participant A, right SCC stimulation significantly decreased delta power (adj.*p* < 0.05) but no significant difference was observed between SCC and VC/VS stimulation in delta power.

## 4 Discussion

**FIGURE 6:**
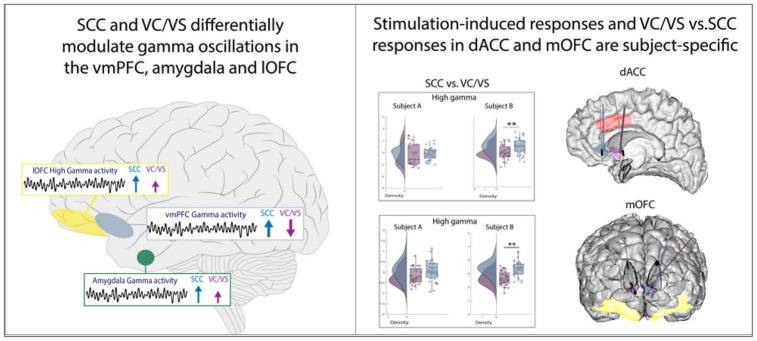
Summary of results.

Through a unique intracranial stimulation and recording dataset collected in two participants with TRD, we obtained results with two main conclusions (Figure 6). First, we demonstrate that two canonical targets for psychiatric neuromodulation, the SCC and VC/VS, elicit network-wide neurophysiological responses in both high and low frequency activity following stimulation. Second, as hypothesized, we show that stimulation in the SCC and VC/VS drive differentiable neural responses. Specifically, we observed opposite effects on gamma activity in the vmPFC, and differing degrees of modulation on gamma activity in the lOFC and amygdala, where SCC stimulation consistently drives a greater increase in gamma oscillations relative to the VC/VS.

Previous tractography work demonstrates that projections from the SCC and VC/VS overlap in the amygdala and medial PFC, but the anatomical trajectory and pattern of connectivity of these projections are distinct (Gutman et al., 2009; Zhu et al., 2021), including the sub-regions that receive projections from the SCC and VC/VS, respectively (Zhu et al., 2021). The difference in connectivity patterns may partially account for the distinct patterns of gamma activity in the vmPFC and amygdala between the two stimulation targets. The modulation of gamma activity seen in the amygdala is additionally supported by a recent study implementing amygdala gamma power as a biomarker for closed-loop VC/VS DBS in a case study for TRD (Scangos et al., 2021a). Interestingly, our results also show modulation of gamma activity in the amygdala following stimulation in *both* targets, but to differing degrees.

Previous studies have also demonstrated anatomical connectivity between the SCC and the OFC, and the VC/VS and the OFC (Johansen-Berg et al., 2008; Haber, 2016), and initial results from our group have shown differing effective connectivity between the SCC and VC/VS to the lOFC, respectively, in TRD participants (Adkinson et al., 2022), leading us to expect differences in neural responses between VC/VS and SCC stimulation. While we observed differing degrees of gamma power modulation in the lOFC depending on the DBS target stimulation in both participants, we did not always observe an overlap in neural responses to stimulation between lOFC and mOFC which might be explained in part by previous work indicating the distinct roles of the lateral vs. medial OFC (Cheng et al., 2016; Rao et al., 2018).

While both VC/VS and SCC stimulation can ameliorate depressive symptoms, they have been described to modulate different dimensions of affective processing and mood: VC/VS stimulation has been reported to increase motivation and energy (Malone et al., 2009; Gibson et al., 2017), while SCC stimulation has reportedly increased calmness, alertness and exteroceptive awareness (Choi et al., 2015; Riva-Posse et al., 2019). It is possible that the dissociable increase/decrease in gamma activity in the vmPFC and the differing degree of gamma modulation in the amygdala and lOFC may be underlying the differences in SCC vs. VC/VS stimulation described in acute behavioral reports seen elsewhere and further work will elucidate our understanding of this phenomenon.

In the dACC, we expected consistent differences in gamma power modulation following SCC and VC/VS stimulation, given recent work implicating dACC gamma power in positive affective behaviors (Bijanzadeh et al., 2022) and differential connectivity of the dACC to the VC/VS (Haber, 2016; Baldermann et al., 2021) and SCC (Johansen-Berg et al., 2008). However, differences in gamma responses between SCC and VC/VS stimulation were participant-specific. Additionally, within a given DBS lead, we observed participant-specific responses between post-stim and pre-stim in the dACC, as well as all other ROIs. The observed participant-specific stimulation responses both between and within the VC/VS and SCC suggest that increasing efforts to personalize therapy may rely on these within-participant electrophysiological signatures across networks to deliver optimized stimulation. Efforts utilizing participant-specific biomarkers for psychiatric DBS have been recently successfully demonstrated (Scangos et al., 2021a), alongside network-guided neuromodulation for psychiatric disorders (Cole et al., 2022).

Future efforts incorporating behavioral measures corresponding to functional domains within mental illness, alongside electrophysiological measurements to build brain-behavior relationships with stimulation will help identify generalizable principles that can be potentially extended to sub-domains of MDD in the broader population (Allawala et al., 2021)**. Even in the absence of behavioral measurements, characterization of stimulation-induced neural response during resting state, especially between DBS targets, enables tailoring of therapy (i.e., selecting an optimal DBS target and stimulation paradigm) for disorders such as depression, based on a patient’s neural response to stimulation, putative neural biomarker of mood, or another functional domain.** Our study methods currently largely operate as a research tool, and while subject-specific differences are observable, it is surmisable that a greater level of consistency is achievable across a larger cohort of participants as we build on a higher sample size to quantify and model network responses to stimulation, and improve our understanding of biomarkers of symptoms in TRD. For example, as more established neural biomarkers of symptom severity (Xiao et al., 2023) or dysfunctional affective or cognitive processing in patients with TRD are uncovered, some degree of personalization and optimization would be feasible with stimulation across the VC/VS and SCC DBS targets. Indeed, efforts utilizing biomarkers to understand optimal intervention have been recently successfully demonstrated (Alagapan et al., 2023), and network-guided neuromodulation to treat psychiatric disorders has gained traction in recent years. An example of the latter is intermittent theta-burst TMS to treat depression, which currently utilizes resting state functional connectivity between the neuromodulation target of interest for TMS (dlPFC) and the SCC (an extant DBS target) to determine the sub-region for stimulation targeting (Cole et al., 2022), and to predict treatment response outcomes with TMS in the field of non-invasive neuromodulation.

Current-steered DBS provides an added parameter for differentially modulating implicated networks and neural biomarkers. A finer grained approach to precisely target anatomical regions implicated in psychopathology of depression for a desired behavioral responses is needed (Figee et al., 2022), and the degree of stimulation-induced neural response may plausibly determine a patient’s therapeutic response. Future work with a larger number of stimulation trials and participants is needed to understand the extent of the effect that directionality may have on connectivity across prefrontal and limbic networks.

Primary limitations of this study include the small sample size (*N* = 2), and lack of randomization of stimulation conditions within or across the two DBS targets. While a rigorous pipeline for optimal surgical targeting was implemented (Sheth et al., 2021), one possible reason for inconsistent results within and across participants is that small variations in targeting may result in modifications in the electrophysiological effects observed. As we were concerned about insufficient time for stimulation washout between trials and stimulation parameters, the baseline window used for analysis was a 5-min recording collected prior to stimulation experiments. To address possible temporal autocorrelation in the baseline recording, we performed a correction procedure (Supplementary Figure 2). However, delta power in vmPFC and lOFC in Participant B had a larger amount of autocorrelation during the baseline recording that could not be fully corrected with our approach, thus, results for those specific neural features must be viewed provisionally.

## Data availability statement

The raw data supporting the conclusions of this article will be made available by the authors, without undue reservation.

## Ethics statement

The studies involving humans were approved by the Baylor College of Medicine Institutional Review Board. The studies were conducted in accordance with the local legislation and institutional requirements. The participants provided their written informed consent to participate in this study.

## Author contributions

AA: Conceptualization, Data curation, Formal analysis, Funding acquisition, Investigation, Methodology, Project administration, Visualization, Writing – original draft, Writing – review and editing, Software, Validation. KB: Data curation, Supervision, Writing – review and editing. DO: Resources, Writing – review and editing, Data curation, Investigation, Software. RM: Visualization, Writing – review and editing, Data curation. JA: Data curation, Writing – review and editing. VP: Writing – review and editing, Data curation. BS: Writing – review and editing, Data curation, Resources. MR: Data curation, Writing – review and editing, Software. SM: Writing – review and editing, Supervision. MH: Writing – review and editing, Supervision, Formal analysis, Methodology, Validation. WG: Writing – review and editing, Supervision, Funding acquisition. NP: Investigation, Resources, Writing – review and editing, Writing – original draft, Funding acquisition, Methodology, Project administration, Supervision. SS: Data curation, Resources, Writing – review and editing, Conceptualization, Funding acquisition, Investigation, Supervision, Visualization, Writing – original draft, Methodology, Project administration. DB: Funding acquisition, Methodology, Supervision, Visualization, Writing – original draft, Writing – review and editing, Investigation.
