# Supplementary material for: Prefrontal network engagement by deep brain stimulation in limbic hubs
Source: Front Hum Neurosci. 2024 Jan 12;17:1291315. doi: 10.3389/fnhum.2023.1291315 (PMC10813208; doi:10.3389/fnhum.2023.1291315)
Supplement: Supplementary file 1 [file Data_Sheet_1.docx]

**Prefrontal network engagement by deep brain stimulation in limbic hubs**

Allawala et al.

**Supplementary Figures**





**Supplementary Figure S1:** Location of sEEG and DBS electrodes and tested stimulation configurations in Subject B.

(A - B) Anatomical locations of sEEG contacts and DBS leads within Subject B. Both subjects had recording sEEG contacts across the vmPFC, dlPFC, dACC, OFC and Temporal cortex. (C) Contact configurations tested on each DBS lead. A total of 7 current configurations were tested across each DBS lead, resulting in 35 trials of stimulation per lead.

**
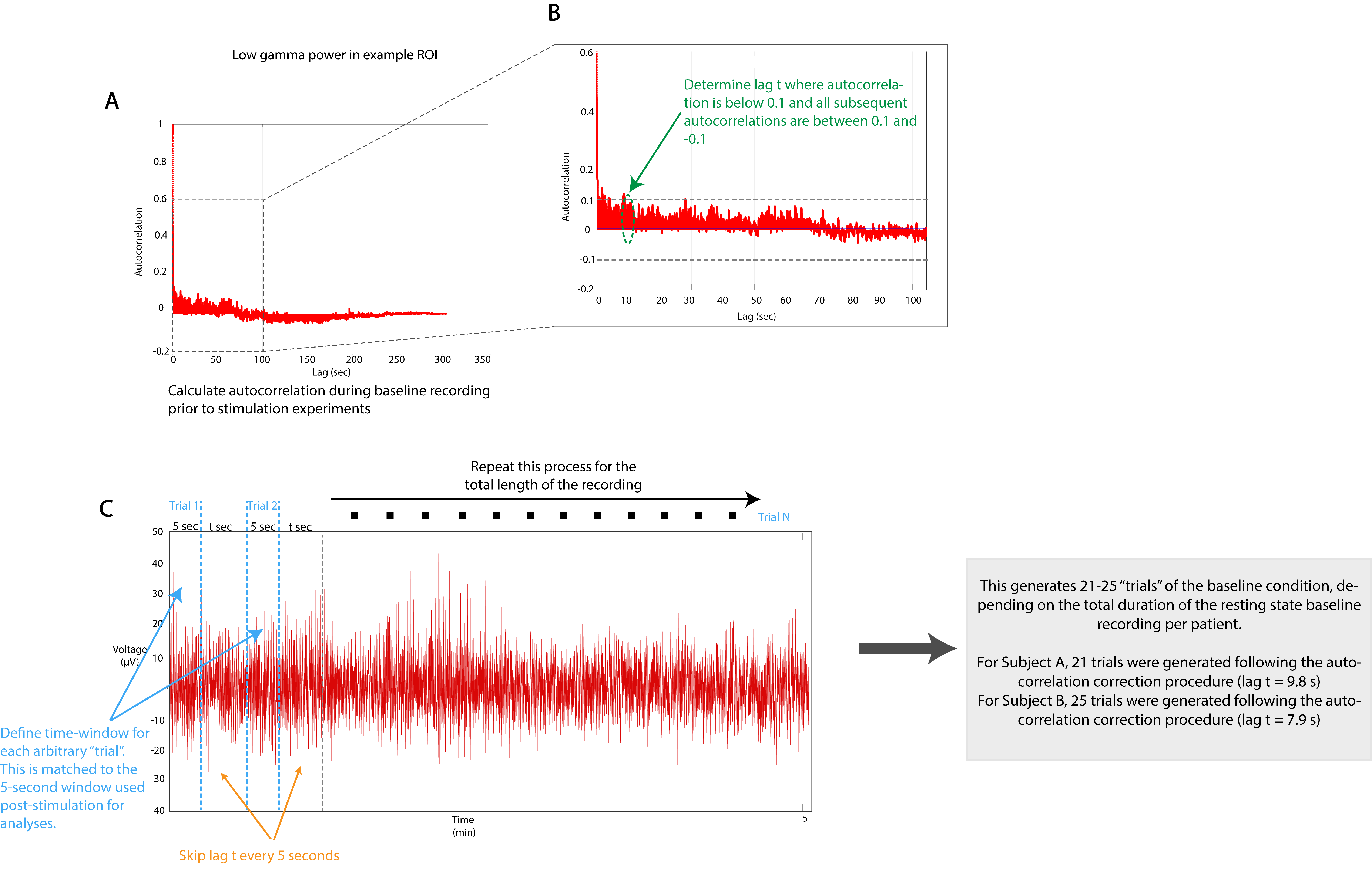
Supplementary Figure S2:** Autocorrelation correction procedure for baseline recordings.

We performed a correction procedure to account for any autocorrelation in our baseline recordings. We took 5-second windows (to match the length of time for each trial in the post-stimulation data) and skipped t seconds between them to allow for any existing autocorrelation to disappear, and so that we could model surrogate “trials” as independent (which is necessary for permutation testing). In order to choose the appropriate lag t, we performed the following steps: we first computed power spectral estimates across the entire recording, and using the autocorr.m function in MATLAB, calculated the autocorrelation for each region of interest (ROI) and frequency band of interest within each subject. For example, autocorrelation values during the baseline recording of low gamma power in an example ROI for Subject A is shown in (A). We then identified a lag t for which the autocorrelation is below 0.1 and all subsequent autocorrelation values are below 0.1 and greater than -0.1 for all frequency bands and ROIs (indicated by dashed lines in (B)). Additionally, for each subject we ensured that lag t that was larger than the majority of the lags identified across all regions and frequency bands, ensuring that the number of samples is the same across all ROIs and neural features. We observed a few exceptions in a few neural features and ROIs where this procedure could not account for autocorrelation – in these cases the autocorrelations were extended for such long lags that we would have to discard a significant amount of data. In such cases, we still used our lag t for these exceptions and results for these exceptions can be viewed provisionally. The exceptions are listed as follows: delta band in vmPFC and lOFC in subject B. The steps outlined in A-C are performed for each subject separately.

**Supplementary Methods**

*Participant Information*

Subject A was a 37 year old male Hispanic patient comorbid with generalized anxiety disorder. Medications taken at the time of enrolment in the current study included Tianeptine and Diphenhydramine.

Subject B was 57 year old female Caucasian patient comorbid with generalized anxiety disorder and panic disorder. Medications taken at the time of enrolment in the current study include Fluoxetine, Buspirone, Clonazepam, Zolpidem, Sumatriptan, Rosuvastatin and Lisinopril.

*Surgical Implantation*

We leveraged an existing well-established approach of sEEG monitoring routinely used for the understanding of epileptic networks for use in TRD. The sEEG monitoring platform has rarely been used for disorders besides epilepsy, and in our study functions as a tool for individualized network analysis in TRD, with the goal of enabling personalized and optimizable delivery of therapeutic DBS and increase the likelihood of lasting symptom alleviation.

The initial surgery consisted of intracranially implanting 4 DBS leads (bilateral SCC and VC/VS) and 10 sEEG electrodes. Target locations for both sEEG electrodes and DBS leads were determined based on high-resolution structural connectivity imaging. DBS leads were positioned to span the region of SCC and VC/VS with the joint maximal probability of connectivity with white matter tracts critically associated with each respective target and DBS leads were externalized to provide access for stimulation delivery.

This study additionally incorporated a holographic augmented reality platform that enabled interactive visualization of the DBS and sEEG targets and white matter tracts connecting them, alongside traditional stereotactic planning tools. The holographic augmented realist platform created a collaborative planning environment and provided a three-dimensional perspective of the extended networks that were targeted with this approach.

*Outpatient Stimulation*

During the outpatient phase during which open-loop chronic stimulation was delivered, DBS was always delivered bilaterally to the SCC and the VC/VS for Participant A and B. During the randomized double-blinded discontinuation phase, stimulation delivery was discontinued across the VC/VS or the SCC (randomly selected). The efficacy of stimulation during the open-loop and blinded discontinuation phase are reported in Sheth et al. 2021 for Participant A. For Participant B and onwards, details on clinical outcomes will be reported in a separate manuscript.

*Statistical Analysis*

The permutation testing procedure and single-step maxT procedure is described in detail as follows:

Let $M$ be a $d\times n$ matrix where $M_{ij}$ denotes the measurement of the $i$th feature (e.g., $\alpha$-power on recording electrode 3) on the $j$th trial. Let $C$ be a $1\times n$ vector where $C_{j}\in\{1,2\}$ denotes which of two conditions the $j$th trial corresponds to (e.g., pre-stim $=1$, post-stim $=2$). $M$ is held fixed throughout, so we suppress it in our notation, but all of the statistics defined below operate on $M$. We do not suppress $C$ in our notation, since we will be permuting the entries of $C$ for the permutation tests described below. Let $T(C)$ be a $d\times1$ vector where the $i$th entry $T_{i}(C)$ is a statistic for comparing trials in condition $1$ to trials in condition $2$ as indicated by $C$ on the basis of the $i$th feature measured in $M$. In the following our statistic will be the absolute value of the classical test-statistic used in the two-sample (unpaired) t-test assuming equal variances, namely,

$$T_{i}\left( C \right)=\frac{\left| \hat{\mu}_{i1}\left( C \right)-\hat{\mu}_{i2}(C) \right|}{\sqrt{\left( \frac{1}{n_{1}}+\frac{1}{n_{2}} \right)\left( \frac{{(n}_{1}-1)s_{i1}^{2}(C)+\left( n_{2}-1 \right)s_{i2}^{2}(C)}{n-2} \right)}}$$

where $n_{b}$ is the number of trials with condition $b\in\{1,2\}$, namely,

$$n_{b}=\#\{j:C_{j}=b\}$$

where $\hat{\mu}_{ib}$ is the sample mean of the measurements of the $i$th feature in condition $b\in\{1,2\}$, namely,

$$\hat{\mu}_{ib}(C)=\frac{1}{n_{b}}\sum_{j:C_{j}=b} M_{ij}$$

and where $s_{ib}^{2}$ is the sample variance of the measurements of the $i$th feature,

$$s_{ib}^{2}\left( C \right)=\frac{1}{n_{b}-1}\sum_{j:C_{j}=b} \left( M_{ij}-\hat{\mu}_{ib} \right)^{2}$$

For clarity, we note that $n_{1}+n_{2}=n$ and that both $n_{1}$ and $n_{2}$ depend on $C$, but not on the order of the entries in $C$, so we have suppressed that dependence in our notation.

If $\pi=\left( \pi_{1},\ldots,\pi_{n} \right)$ is a permutation of the numbers $(1,\ldots,n)$, then we define the $1\times n$ vector of permuted condition labels $C^{\pi}$ via $C_{j}^{\pi}=C_{\pi_{j}}$. If $\pi$ is a random permutation (chosen uniformly from all possible $n!$ permutations), then $T_{i}(C^{\pi})$ uses the above two-sample t-test statistic to compare trials randomly assigned to condition $1$ to trials randomly assigned to condition $2$ on the basis of the measurements of feature $i$. The vector $T\left( C^{\pi} \right)$ contains the results of this comparison for each of the $d$ features. Importantly, each entry of $T(C^{\pi})$ uses the *same* permutation $\pi$.

Consider the null hypothesis $H_{i}$ that the measurements of the $i$th feature are unrelated to the condition labels in $C$. (More formally, the null hypothesis is that $M_{i1},\ldots,M_{in}$ are conditionally exchangeable given $C$.) A Monte Carlo permutation test can be used to create valid p-values for each of the null hypotheses $H_{1},\ldots,H_{d}$ as follows. We sample a total of $L$ independent random permutations $\pi^{k}=(\pi_{1}^{k},\ldots,\pi_{n}^{k})$ for $k=1,\ldots,L$. We use $L=1000$ in the paper. For each such permutation we compute the t-statistic vector $T^{k}=T(C^{\pi^{k}})$. We also compute the t-statistic vector for the original $C$, namely, $T^{0}=T(C)$. A valid p-value for $H_{i}$ is

$$p_{i}=\frac{1}{L+1}\sum_{k=0}^{L} \mathbb{1}\left[ T_{i}^{k}\geq T_{i}^{0} \right]$$

where the indicator function $\mathbb{1}[A]$ returns $1$ if $A$ is true and $0$ if $A$ is false. This p-value gives the fraction of times (among all permutations and the original) that the test statistic for feature $i$ is at least as large as the original observed test statistic for feature $i$. It is important that the $k=0$ case is included in the sum and that we use $\geq$ and not $>$ in the comparison to ensure the validity of the test. Note that the smallest possible value of $p_{i}$ is $1/(L+1)$. These $p_{i}$ are the uncorrected or unadjusted p-values used in the paper.

To control for multiple hypothesis tests, we use a single-step version of the max-T procedure that will strongly control the familywise error rate (FWER) across all features subject to a minor caveat described below. For the $k$th permutation (including the case $k=0$ corresponding to no permutation), define the maximum statistic as

$$T_{*}^{k}=\max_{i=1,\ldots,d} T_{i}^{k}$$

which is a number, not a vector. A valid adjusted p-value for $H_{i}$ that controls the FWER over the collection $H_{1},\ldots,H_{d}$ is

$$p_{i}^{*}=\frac{1}{L+1}\sum_{k=0}^{L} \mathbb{1}\left[ T_{*}^{k}\geq T_{i}^{0} \right]$$

This adjusted p-value gives the fraction of times (among all permutations and the original) that the maximum test statistic over all features is at least as large as the original observed test statistic for feature $i$. We always have $p_{i}^{*}\geq p_{i}$, but equality is definitely possible (unlike, say, Bonferroni adjusted p-values). These $p_{i}^{*}$ are the corrected or adjusted p-values used in the paper. Our rationale for using max-T adjusted p-values instead of Bonferroni adjusted p-values is that max-T adjusted p-values can have much better power for positively correlated p-values (which we expect to see in our context) and also require many fewer permutations to achieve good power.

Unlike the Bonferroni correction, the max-T procedure requires a technical assumption called *subset pivotality* to ensure strong control of the FWER. Roughly speaking, the subset pivotality assumption states that the joint distribution of all features where the null hypothesis is true does not change across conditions. Consider, for instance, the following scenario. The distribution of feature $1$ is unchanged between condition $1$ and condition $2$ so that $H_{1}$ is true. Similarly, the distribution of feature $2$ is unchanged between condition $1$ and condition $2$ so that $H_{2}$ is true. But features $1$ and $2$ are more correlated in condition $1$ than in condition $2$. This situation violates the subset pivotality assumption, since the joint distribution of two or more features is changing across conditions while the marginal distributions of each of these features remains the same. In a situation like this it is possible that either $H_{1}$ or $H_{2}$ (or both) would be rejected more frequently than expected by chance using the max-T adjusted p-values.

There is no way to know if the subset pivotality assumption is accurate in our context, although we note that it is a strikingly singular manipulation to modify the joint distribution of a collection of variables with absolutely no modification of the marginal distributions of the variables. (In fact, it is impossible to do so if one also requires that the distributions of linear combinations of the variables remain unchanged.) For our scientific purposes, however, we think that the risk of violations of subset pivotality are small for the following reasons.

(1) The primary conclusion of our paper is that there are detectable electrophysiological differences between DBS used in different brain regions and/or using different stimulation parameters. This conclusion is unaffected by the subset pivotality assumption. Indeed, consider the global p-value

$$p_{*}=\frac{1}{L+1}\sum_{k=0}^{L} \mathbb{1}\left[ T_{*}^{k}\geq T_{*}^{0} \right]$$

This is simply a Monte Carlo permutation test p-value for comparing condition $1$ versus condition $2$ on the basis of the test statistic $T_{*}$, which uses all of the features. Since we have $p_{*}\leq p_{i}^{*}$ for all $i=1,\ldots,d$, then a rejection of any $H_{i}$ based on the adjusted p-value $p_{i}^{*}$ automatically implies a rejection of the null hypothesis that there is no difference between conditions.

(2) If the subset pivotality condition is not true, the only danger is that we will mistakenly conclude that differences in DBS create differences in some brain region (or frequency band) when, in fact, DBS only creates differences in that region’s (or band’s) relationship to another region (or band). It begins to border on philosophical whether this is even a mistaken scientific conclusion or not.

(3) Furthermore, since $p_{i}^{*}\geq p_{i}$ for each feature $i$, our adjusted p-values can only be used to reject null hypothesis $H_{i}$ when the data provide strong evidence against $H_{i}$ when viewed in isolation, i.e., when the $i$th feature measurements are noticeably different across conditions.

**Supplementary Tables**

| **Subject A** | | | | | | |
| --- | --- | --- | --- | --- | --- | --- |
| ROIs | Frequency | | | | | |
|  | Delta | Theta | Alpha | Beta | Low Gamma | High Gamma |
| dACC | 0.106 | 1.000 | 0.657 | 0.179 | 0.468 | 1.000 |
| Amygdala | 0.983 | 0.087 | 0.035 | 0.001 | 0.001 | 0.001 |
| mOFC | 0.123 | 0.182 | 0.001 | 0.019 | 0.229 | 0.190 |
| lOFC | 1.000 | 0.860 | 0.130 | 0.001 | 0.019 | 0.156 |
| vmPFC | 0.001 | 0.609 | **0.007** | 0.001 | 0.171 | 0.008 |
| **Subject B** | | | | | | |
| ROIs | Frequency | | | | | |
|  | Delta | Theta | Alpha | Beta | Low Gamma | High Gamma |
| dACC | 0.783 | 1.000 | 0.920 | 0.014 | 0.002 | 0.001 |
| Amygdala | 0.444 | 0.041 | 1.000 | 1.000 | 0.001 | 0.001 |
| mOFC | 0.663 | 0.147 | 0.787 | 0.999 | 0.132 | 0.032 |
| lOFC | 0.626 | 0.608 | 0.667 | 0.804 | 1.000 | 0.050 |
| vmPFC | 0.235 | 1.000 | 1.000 | 1.000 | 1.000 | 0.872 |

**Supplementary Table S1****:** Corrected p-values resulting from statistical testing between stimulation in the left SCC and baseline (pre-stim/baseline vs post-stimulation)

| **Subject A** | | | | | | |
| --- | --- | --- | --- | --- | --- | --- |
| ROIs | Frequency | | | | | |
|  | Delta | Theta | Alpha | Beta | Low Gamma | High Gamma |
| dACC | 0.021 | 1.000 | 1.000 | 0.553 | 0.999 | 1.000 |
| Amygdala | 1.000 | 0.763 | 0.001 | 0.001 | 0.001 | 0.001 |
| mOFC | 0.440 | 0.560 | 0.001 | 0.001 | 0.165 | 0.136 |
| lOFC | 0.997 | 0.964 | 0.710 | 0.001 | 0.001 | 0.007 |
| vmPFC | 0.001 | 0.266 | 0.038 | 0.001 | 1.000 | 0.314 |
| **Subject B** | | | | | | |
| ROIs | Frequency | | | | | |
|  | Delta | Theta | Alpha | Beta | Low Gamma | High Gamma |
| dACC | 0.511 | 1.000 | 1.000 | 0.003 | 0.108 | 0.001 |
| Amygdala | 0.538 | 0.001 | 0.160 | 0.005 | 0.001 | 0.001 |
| mOFC | 0.918 | 0.230 | 1.000 | 0.359 | 1.000 | 0.993 |
| lOFC | 0.918 | 0.572 | 1.000 | 0.368 | 1.000 | 0.751 |
| vmPFC | 0.068 | 0.951 | 0.822 | 0.292 | 1.000 | 1.000 |

**Supplementary Table S2:** Corrected p-values resulting from statistical testing between stimulation in the right SCC and baseline (pre-stim/baseline vs post-stimulation).

| **Subject A** | | | | | | |
| --- | --- | --- | --- | --- | --- | --- |
| ROIs | Frequency | | | | | |
|  | Delta | Theta | Alpha | Beta | Low Gamma | High Gamma |
| dACC | 0.020 | 0.945 | 1.000 | 1.000 | 0.917 | 1.000 |
| Amygdala | 0.969 | 0.308 | 0.948 | 0.593 | 0.063 | 0.018 |
| mOFC | 0.038 | 0.005 | 0.004 | 0.870 | 1.000 | 1.000 |
| lOFC | 0.565 | 0.079 | 0.100 | 1.000 | 0.152 | 0.994 |
| vmPFC | 0.002 | 0.070 | 1.000 | 1.000 | 0.824 | 0.762 |
| **Subject B** | | | | | | |
| ROIs | Frequency | | | | | |
|  | Delta | Theta | Alpha | Beta | Low Gamma | High Gamma |
| dACC | 0.927 | 0.944 | 0.291 | 0.001 | 0.019 | 0.001 |
| Amygdala | 0.215 | 0.062 | 1.000 | 0.027 | 0.592 | 0.084 |
| mOFC | 0.988 | 0.143 | 0.734 | 1.000 | 1.000 | 0.989 |
| lOFC | 1.000 | 0.870 | 0.999 | 0.995 | 1.000 | 1.000 |
| vmPFC | 0.123 | 1.000 | 0.984 | 1.000 | 0.400 | 0.253 |

**Supplementary Table S3:** Corrected p-values resulting from statistical testing between stimulation in the left VC/VS and baseline (pre-stim/baseline vs post-stimulation).

| **Subject A** | | | | | | |
| --- | --- | --- | --- | --- | --- | --- |
| ROIs | Frequency | | | | | |
|  | Delta | Theta | Alpha | Beta | Low Gamma | High Gamma |
| dACC | 0.856 | 0.023 | 1.000 | 0.965 | 1.000 | 1.000 |
| dlPFC | 1.000 | 0.991 | 0.908 | 0.991 | 0.028 | 0.017 |
| mOFC | 0.016 | 0.614 | 0.994 | 1.000 | 1.000 | 0.999 |
| lOFC | 1.000 | 1.000 | 1.000 | 0.994 | 0.999 | 0.748 |
| vmPFC | 0.004 | 0.023 | 1.000 | 0.863 | 0.215 | 0.961 |
| **Subject B** | | | | | | |
| ROIs | Frequency | | | | | |
|  | Delta | Theta | Alpha | Beta | Low Gamma | High Gamma |
| dACC | 1.000 | 0.884 | 0.662 | 0.003 | 1.000 | 0.001 |
| Amygdala | 0.473 | 0.438 | 1.000 | 0.092 | 0.999 | 0.001 |
| mOFC | 0.745 | 0.965 | 0.971 | 0.005 | 0.454 | 1.000 |
| lOFC | 0.380 | 0.666 | 1.000 | 0.002 | 0.253 | 1.000 |
| vmPFC | 0.855 | 0.997 | 1.000 | 0.001 | 0.029 | 0.575 |

**Supplementary Table S4:** Corrected p-values resulting from statistical testing between stimulation in the right VC/VS and baseline (pre-stim/baseline vs post-stimulation).

| **Subject A** | | | | | | |
| --- | --- | --- | --- | --- | --- | --- |
| ROIs | Frequency | | | | | |
|  | Delta | Theta | Alpha | Beta | Low Gamma | High Gamma |
| dACC | 0.983 | 1.000 | 0.529 | 0.216 | 1.000 | 0.988 |
| Amygdala | 1.000 | 1.000 | 0.489 | 0.058 | 0.024 | 0.046 |
| mOFC | 0.024 | 0.111 | 0.943 | 0.714 | 0.453 | 0.663 |
| lOFC | 0.355 | 0.987 | 1.000 | 0.003 | 0.001 | 0.009 |
| vmPFC | 0.030 | 1.000 | 0.026 | 0.001 | 0.001 | 0.001 |
| **Subject B** | | | | | | |
| ROIs | Frequency | | | | | |
|  | Delta | Theta | Alpha | Beta | Low Gamma | High Gamma |
| dACC | 1.000 | 0.921 | 0.984 | 0.422 | 0.877 | 0.903 |
| Amygdala | 1.000 | 1.000 | 1.000 | 0.038 | 0.001 | 0.001 |
| mOFC | 0.994 | 1.000 | 1.000 | 0.970 | 0.020 | 0.001 |
| lOFC | 0.981 | 1.000 | 0.994 | 1.000 | 1.000 | 0.003 |
| vmPFC | 1.000 | 1.000 | 0.982 | 1.000 | 0.075 | 0.001 |

**Supplementary Table S5:** Corrected p-values resulting from statistical testing between stimulation in the left SCC and stimulation in the left VC/VS (baseline-subtracted post-stimulation windows used for both DBS leads)

| **Subject A** | | | | | | |
| --- | --- | --- | --- | --- | --- | --- |
| ROIs | Frequency | | | | | |
|  | Delta | Theta | Alpha | Beta | Low Gamma | High Gamma |
| dACC | 1.000 | 0.137 | 1.000 | 0.009 | 1.000 | 1.000 |
| Amygdala | 1.000 | 1.000 | 0.620 | 0.002 | 0.002 | 0.001 |
| mOFC | 0.300 | 1.000 | 0.414 | 0.004 | 0.058 | 0.837 |
| lOFC | 0.990 | 0.991 | 0.992 | 0.047 | 0.004 | 0.391 |
| vmPFC | 0.798 | 0.998 | 0.071 | 0.001 | 0.012 | 0.004 |
| **Subject B** | | | | | | |
| ROIs | Frequency | | | | | |
|  | Delta | Theta | Alpha | Beta | Low Gamma | High Gamma |
| dACC | 0.001 | 0.694 | 0.543 | 1.000 | 0.015 | 1.000 |
| Amygdala | 1.000 | 0.010 | 0.264 | 1.000 | 0.001 | 1.000 |
| mOFC | 0.003 | 0.725 | 0.768 | 0.549 | 0.024 | 0.491 |
| lOFC | 0.002 | 1.000 | 1.000 | 0.209 | 0.336 | 0.217 |
| vmPFC | 0.012 | 1.000 | 0.998 | 0.097 | 0.025 | 0.278 |

**Supplementary Table S6:** Corrected p-values resulting from statistical testing between stimulation in the right SCC and stimulation in the right VC/VS (baseline-subtracted post-stimulation windows used for both DBS leads)

**Supplementary Tables with uncorrected p-values following permutation testing**

| **Subject A** | | | | | | |
| --- | --- | --- | --- | --- | --- | --- |
| ROIs | Frequency | | | | | |
|  | Delta | Theta | Alpha | Beta | Low Gamma | High Gamma |
| dACC | 0.006 | 0.660 | 0.052 | 0.006 | 0.029 | 0.361 |
| Amygdala | 0.193 | 0.006 | 0.001 | 0.001 | 0.001 | 0.001 |
| mOFC | 0.006 | 0.012 | 0.001 | 0.002 | 0.015 | 0.009 |
| lOFC | 0.518 | 0.097 | 0.006 | 0.001 | 0.002 | 0.006 |
| vmPFC | 0.001 | 0.048 | 0.002 | 0.001 | 0.008 | 0.001 |
| **Subject B** | | | | | | |
| ROIs | Frequency | | | | | |
|  | Delta | Theta | Alpha | Beta | Low Gamma | High Gamma |
| dACC | 0.073 | 0.994 | 0.158 | 0.002 | 0.001 | 0.001 |
| Amygdala | 0.036 | 0.002 | 0.730 | 0.461 | 0.001 | 0.001 |
| mOFC | 0.054 | 0.007 | 0.096 | 0.378 | 0.011 | 0.004 |
| lOFC | 0.048 | 0.050 | 0.057 | 0.090 | 0.887 | 0.001 |
| vmPFC | 0.009 | 0.733 | 0.846 | 0.600 | 0.819 | 0.119 |

**Supplementary Table S7:** Uncorrected p-values resulting from statistical testing between stimulation in the left SCC and baseline (pre-stim/baseline vs post-stimulation)

| **Subject A** | | | | | | |
| --- | --- | --- | --- | --- | --- | --- |
| ROIs | Frequency | | | | | |
|  | Delta | Theta | Alpha | Beta | Low Gamma | High Gamma |
| dACC | 0.002 | 0.742 | 0.561 | 0.036 | 0.279 | 0.350 |
| Amygdala | 0.342 | 0.072 | 0.001 | 0.001 | 0.001 | 0.001 |
| mOFC | 0.016 | 0.050 | 0.001 | 0.001 | 0.011 | 0.007 |
| lOFC | 0.232 | 0.146 | 0.057 | 0.001 | 0.001 | 0.001 |
| vmPFC | 0.001 | 0.029 | 0.004 | 0.001 | 0.399 | 0.021 |
| **Subject B** | | | | | | |
| ROIs | Frequency | | | | | |
|  | Delta | Theta | Alpha | Beta | Low Gamma | High Gamma |
| dACC | 0.023 | 0.906 | 0.872 | 0.001 | 0.005 | 0.001 |
| Amygdala | 0.031 | 0.001 | 0.007 | 0.002 | 0.001 | 0.001 |
| mOFC | 0.150 | 0.006 | 0.606 | 0.024 | 0.670 | 0.292 |
| lOFC | 0.146 | 0.044 | 0.705 | 0.041 | 0.651 | 0.066 |
| vmPFC | 0.001 | 0.175 | 0.107 | 0.019 | 0.640 | 0.828 |

**Supplementary Table S8:** Uncorrected p-values resulting from statistical testing between stimulation in the right SCC and baseline (pre-stim/baseline vs post-stimulation).

| **Subject A** | | | | | | |
| --- | --- | --- | --- | --- | --- | --- |
| ROIs | Frequency | | | | | |
|  | Delta | Theta | Alpha | Beta | Low Gamma | High Gamma |
| dACC | 0.003 | 0.143 | 0.876 | 0.731 | 0.115 | 0.696 |
| Amygdala | 0.174 | 0.016 | 0.139 | 0.035 | 0.004 | 0.001 |
| mOFC | 0.001 | 0.001 | 0.001 | 0.092 | 0.743 | 0.511 |
| lOFC | 0.034 | 0.001 | 0.005 | 0.692 | 0.014 | 0.242 |
| vmPFC | 0.001 | 0.004 | 0.480 | 0.555 | 0.091 | 0.060 |
| **Subject B** | | | | | | |
| ROIs | Frequency | | | | | |
|  | Delta | Theta | Alpha | Beta | Low Gamma | High Gamma |
| dACC | 0.138 | 0.173 | 0.017 | 0.001 | 0.001 | 0.001 |
| Amygdala | 0.007 | 0.005 | 0.476 | 0.001 | 0.035 | 0.004 |
| mOFC | 0.264 | 0.007 | 0.066 | 0.712 | 0.920 | 0.287 |
| lOFC | 0.390 | 0.113 | 0.368 | 0.329 | 0.622 | 0.903 |
| vmPFC | 0.003 | 0.561 | 0.258 | 0.828 | 0.036 | 0.015 |

**Supplementary Table S9:** Uncorrected p-values resulting from statistical testing between stimulation in the left VC/VS and baseline (pre-stim/baseline vs post-stimulation).

| **Subject A** | | | | | | |
| --- | --- | --- | --- | --- | --- | --- |
| ROIs | Frequency | | | | | |
|  | Delta | Theta | Alpha | Beta | Low Gamma | High Gamma |
| dACC | 0.096 | 0.002 | 0.453 | 0.134 | 0.707 | 0.491 |
| dlPFC | 0.916 | 0.183 | 0.132 | 0.190 | 0.002 | 0.003 |
| mOFC | 0.001 | 0.039 | 0.212 | 0.835 | 0.507 | 0.251 |
| lOFC | 0.582 | 0.737 | 0.358 | 0.222 | 0.229 | 0.063 |
| vmPFC | 0.001 | 0.002 | 0.861 | 0.084 | 0.012 | 0.132 |
| **Subject B** | | | | | | |
| ROIs | Frequency | | | | | |
|  | Delta | Theta | Alpha | Beta | Low Gamma | High Gamma |
| dACC | 0.822 | 0.108 | 0.054 | 0.001 | 0.830 | 0.001 |
| Amygdala | 0.018 | 0.026 | 0.689 | 0.007 | 0.369 | 0.001 |
| mOFC | 0.075 | 0.205 | 0.231 | 0.001 | 0.029 | 0.524 |
| lOFC | 0.020 | 0.055 | 0.465 | 0.001 | 0.012 | 0.720 |
| vmPFC | 0.085 | 0.354 | 0.460 | 0.001 | 0.002 | 0.052 |

**Supplementary Table S10:** Uncorrected p-values resulting from statistical testing between stimulation in the right VC/VS and baseline (pre-stim/baseline vs post-stimulation).

| **Subject A** | | | | | | |
| --- | --- | --- | --- | --- | --- | --- |
| ROIs | Frequency | | | | | |
|  | Delta | Theta | Alpha | Beta | Low Gamma | High Gamma |
| dACC | 0.180 | 0.251 | 0.041 | 0.012 | 0.732 | 0.196 |
| Amygdala | 0.948 | 0.719 | 0.021 | 0.004 | 0.002 | 0.003 |
| mOFC | 0.002 | 0.006 | 0.144 | 0.069 | 0.030 | 0.066 |
| lOFC | 0.019 | 0.208 | 0.955 | 0.001 | 0.001 | 0.001 |
| vmPFC | 0.004 | 0.432 | 0.001 | 0.001 | 0.001 | 0.001 |
| **Subject B** | | | | | | |
| ROIs | Frequency | | | | | |
|  | Delta | Theta | Alpha | Beta | Low Gamma | High Gamma |
| dACC | 0.287 | 0.114 | 0.195 | 0.022 | 0.092 | 0.082 |
| Amygdala | 0.413 | 0.354 | 0.679 | 0.002 | 0.001 | 0.001 |
| mOFC | 0.221 | 0.822 | 0.806 | 0.156 | 0.003 | 0.001 |
| lOFC | 0.193 | 0.563 | 0.221 | 0.374 | 0.325 | 0.001 |
| vmPFC | 0.494 | 0.718 | 0.189 | 0.417 | 0.002 | 0.001 |

**Supplementary Table S11:** Uncorrected p-values resulting from statistical testing between stimulation in the left SCC and stimulation in the left VC/VS (baseline-subtracted post-stimulation windows used for both DBS leads)

| **Subject A** | | | | | | |
| --- | --- | --- | --- | --- | --- | --- |
| ROIs | Frequency | | | | | |
|  | Delta | Theta | Alpha | Beta | Low Gamma | High Gamma |
| dACC | 0.471 | 0.005 | 0.850 | 0.002 | 0.612 | 0.828 |
| Amygdala | 0.367 | 0.426 | 0.056 | 0.001 | 0.001 | 0.001 |
| mOFC | 0.021 | 0.511 | 0.032 | 0.001 | 0.004 | 0.084 |
| lOFC | 0.203 | 0.203 | 0.215 | 0.003 | 0.001 | 0.029 |
| vmPFC | 0.059 | 0.261 | 0.002 | 0.001 | 0.001 | 0.001 |
| **Subject B** | | | | | | |
| ROIs | Frequency | | | | | |
|  | Delta | Theta | Alpha | Beta | Low Gamma | High Gamma |
| dACC | 0.001 | 0.042 | 0.029 | 0.800 | 0.002 | 0.345 |
| Amygdala | 0.934 | 0.002 | 0.017 | 0.379 | 0.001 | 0.729 |
| mOFC | 0.001 | 0.059 | 0.081 | 0.039 | 0.002 | 0.027 |
| lOFC | 0.001 | 0.625 | 0.619 | 0.012 | 0.025 | 0.011 |
| vmPFC | 0.001 | 0.593 | 0.258 | 0.003 | 0.002 | 0.015 |

**Supplementary Table S12:** Uncorrected p-values resulting from statistical testing between stimulation in the right SCC and stimulation in the right VC/VS (baseline-subtracted post-stimulation windows used for both DBS leads)
